# Nutritional status of children under five years and associated factors in 24 districts of Burkina Faso

**DOI:** 10.1371/journal.pgph.0001248

**Published:** 2023-07-31

**Authors:** T. Bernadette Picbougoum, M. A. Serge Somda, S. Henri Zango, Julia Lohmann, Manuela De Allegri, Hamadou Saidou, Hervé Hien, Nicolas Meda, Annie Robert

**Affiliations:** 1 Institut National de Santé Publique (INSP)/ Centre MURAZ, Bobo-Dioulasso, Burkina Faso; 2 Institut de Recherche Expérimentale et Clinique, Pôle Epidémiologie et Biostatistique, Université catholique de Louvain (UCLouvain), Brussels, Belgium; 3 Université Nazi Boni, Unité de Formation et de Recherche/Sciences et Technique, Bobo-Dioulasso, Burkina Faso; 4 Institut de Recherche en Sciences de la Santé (IRSS), Direction Régionale du Centre-Ouest, Nanoro, Burkina Faso; 5 Department of Global Health and Development, London School of Hygiene & Tropical Medicine, London, United Kingdom; 6 Institute of Global Health, Heidelberg University Hospital and Medical Faculty, Heidelberg, Germany; 7 Heidelberg Institute of Global Health, University Hospital and Medical Faculty, Heidelberg University, Heidelberg, Germany; 8 The World Bank, Yaoundé, Cameroon; 9 Institut de Recherche en Sciences de la Santé (IRSS), Direction Régionale de l’Ouest, Bobo-Dioulasso, Burkina Faso; 10 Université Joseph Ki-Zerbo, UFR/SDS, Ouagadougou, Burkina Faso; Al-Bayan University, IRAQ

## Abstract

Malnutrition in children is a serious health problem, especially in Sub-Saharan Africa, with heavy socioeconomic burdens. The prevalence of stunting remains high in Burkina Faso. There is a need to further investigate undernutrition and identify the major factors contributing to its persistence. We aimed to assess the nutritional status of children aged under five years and the associated factors of undernutrition in Burkina Faso. We conducted a second study using a baseline household survey of the impact assessment of a performance-based financing program. The analysis focused on data of 10,032 children aged 0–59 months collected from households in 537 villages. Anthropometric indicators were assessed using the World Health Organization standards, and their association with children, mothers, and households’ characteristics were assessed using logistic regression. Stunting occurred in 40.1% of children, wasting in 25.1%, and underweight in 34%. Children having both stunting, wasting, and underweight were 7.3%. Stunting and underweight was associated with the sex. Stunting was associated with ethnic groups: Fulani with AdjOR = 1.20 (95%CI: 1.01–1.42), household economic level: poorest AdjOR = 1.25 (95%CI: 1.10–1.43), two and more children aged under five years in households: AdjOR = 1.16 (95%CI: 1.05–1.27), distance more than 5km from household to health facility: with Adj OR = 1.21 (95%CI: 1.10–1.35) and household food insecurity. This study identified the modifiable factors that determine the high prevalence of undernutrition in Burkina Faso. Strategies and interventions to improve the health and economic status of the community are needed to reduce the occurrence of undernutrition.

## Introduction

Childhood malnutrition is a major global health problem that contributes to childhood morbidity, mortality, impaired intellectual development, suboptimal adult work capacity, and increased risk of diseases in adulthood [1]. Globally, malnutrition in all forms (undernutrition, micronutrient deficiencies, overweight, and obesity) remains a challenge. Although it is not yet possible to fully account for the impact of the coronavirus disease (COVID-19) pandemic due to data limitations, in 2020, it was estimated that 22.0 percent (149.2 million) of children under 5 years of age were affected by stunting, 6.7 percent (45.4 million) were suffering from wasting, and 5.7 percent (38.9 million) were overweight [2]. Most children under five years of malnutrition live in Africa or Asia [2]. The economic loss attributable to malnutrition has been estimated to be US$ 3.5 trillion annually, equal to 11 percent of the gross domestic product (GDP) of Africa and Asia [3].

In Burkina Faso, the prevalence of wasting and stunting in children under five years old was estimated in 2010 at 16% and 35%, respectively. More than 35% of deaths in children are caused by malnutrition [4]. Several factors have contributed to this situation such as parasitic and infectious diseases, inadequate feeding during childhood, unsuitable food and dietary practices, inadequate hygiene and sanitation conditions, low quality of health care and education, and poor socioeconomic conditions [5].

Several studies have assessed factors associated with childhood undernutrition at the individual or community level in low-income countries, including Sub-Saharan Africa. Key factors are being male, at the age of 6 to 23 months, longer breastfeeding duration, low birth weight, living in rural areas, parents’ low socioeconomic status and poor education, mother’s BMI, low access to hygiene and sanitation (safe water, hygienic toilet), a large number of members in the household, and environmental factors [6–23]. A handful of studies have focused on the determinants of undernutrition [24–29]. These determinants often differ from one study to another depending on the context of the study. Our study therefore aimed to contribute to strengthen knowledge by assessing the nutritional status of children under five years of age and the associated factors of undernutrition in households. Using data from a comprehensive, quasi-representative household survey conducted in 2014, we examined the prevalence and sociodemographic and economic factors associated with three forms of undernutrition in children under the age of five 5 years old (U5) in Burkina Faso.

## Materials and methods

### Study design

The present study used data from a baseline household survey conducted in the context of the impact evaluation of the Performance-Based Financing (PBF) program in Burkina Faso [30]. Details of this study have been published elsewhere [31–33]. In brief, a household survey was carried out from October 15, 2013, to March 15, 2014, to provide basic indicators for assessing the impact of the PBF initiative on the quality and use of maternal and child health services. The study included villages in six health regions of Burkina Faso, namely Boucle du Mouhoun, Centre-Nord, Centre-Ouest, Nord, Sud-Ouest, and Centre-Est. Twenty-four districts (Nouna, Solenzo, Kongoussi, Kaya, Koudougou, Sapouy, Gourcy, Ouahigouya, Batié, Diébougou, Ouargaye, Tenkodogo, Boromo, Toma, Barsalogho, Ziniaré, Nanoro, Réo, Yako, Boussé, Dano, Gaoua, Zabré and Manga) participated in this study. The survey was conducted among households in these health facilities areas.

### Sampling and study participants

The household survey was conducted in one or two villages selected randomly in the catchment areas of all or a random sample of primary health facilities in each of the 24 districts. Households were selected using a three-stage sampling method. First, clusters were defined in catchment areas of primary healthcare facilities (Centre de Santé et Promotion Sociale (CSPS)). In half of the districts (PBF intervention districts), each CSPS was considered whereas, in the other half of the districts (districts serving as controls in the PBF impact evaluation), a random sample of health facilities was selected, resulting in a total sample of 537 primary health care facilities. Second, one village is randomly selected from each cluster. Third, 15 households were randomly selected in each village for interviews among all households that met the inclusion criteria, namely, at least one woman currently pregnant or having completed pregnancy in the prior two years. Specifically, the listing procedure enabled the identification of all households in the village and asserted whether they fulfilled the inclusion criteria. When more than 15 households were eligible for inclusion, a random sample was drawn. This three-stage process resulted in 7,978 households being randomly selected for inclusion in the survey. The questionnaire was directed at the heads of households, pregnant women, women who had given birth in the last two years at the time of the study, and children under five years old, investigating socio-demographic, economic, and health characteristics. Malaria infection and anemia were diagnosed in all children under five years of age, in pregnant women, and in women who delivered in the last two years at the time of the study and who were present in the household during the visit. Anthropometric data, such as weight, height, and mid-upper arm circumference (MUAC) were also collected.

We used a sub-sample of 10,032 children for whom we had complete and plausible data for all relevant variables, from a total of 12,116 children under five years included in the original household survey sample. Regarding the latter, we considered implausible and excluded children for whom variables, such as age, sex, weight, and length/height, were not fully reported. Subsequently, the selection process considered mothers who had the variables chosen for our study.

### Study variables

The outcome variables were three indicators of nutritional status: stunting, wasting, and underweight in children under five years. Each of these indicators measures different aspects of the nutritional status of children. Stunting is a measure of chronic nutritional deficiency, wasting indicates acute nutritional deficiency, and underweight is a measure of both acute and chronic status. These indicators were assessed by calculating height-for-age, weight-for-height, and weight-for-age indices. Z-scores were used to express the anthropometric standard deviation in line with the Waterlow definitions and the World Health Organization (WHO) 2006 standard [34]. Children were classified as wasted, stunted, or underweight when z-score < -2, moderate when z-score < -2 and ≥ -3, and severe when z-score < -3. Weight-for-height z-scores also allow the identification of overweight and obesity, but we did not include these indicators in our analysis.

The investigated explanatory factors included the sex of the child (male or female), place of residence, ethnic group, mother’s age, educational attainment, household wealth, number of children under five years in the household, and distance from the household to the nearest health facility. Ethnic groups were grouped into three categories according to their regional and behavioral proximity: (1) Moose, (2) Dioula, and similar groups (Bobo, Dagara, Lobi, Senoufo, Bwaba/Dafing, Dafing, and Samo); (3) Fulani and similar groups (Fulfuldé, Gourmantché and Touareg/Bella), and (4) others (Gouroussi, Bissa, and others). Mothers’ educational attainment was classified into three levels: no schooling, primary school, or secondary and higher school. Household wealth was assessed using the standard multiple component analysis (MCA) method to create a wealth index based on the following assets and classify households into quintiles based on housing (type of building, number of rooms, water, and energy supply), assets (TV, radio, fridge, etc.), house, fields, and animals owned. From health facilities and households’ global positioning system (GPS) coordinates, the distance of each household to a health facility was calculated and classified into three categories: under 2 km, between 2 and 5 km, and over 5 km. Finally, classification of households into the country’s climatic areas (Sahelian, Sudano-Sahelian, and Sudanese) and respective food security status were based on national data reports (food security = < 10%, limit food insecurity = 10% - 20%, moderate insecurity = 20% - 30%, severe insecurity > 30%) [35].

Malaria infection and anemia assessed using the Malaria Rapid Diagnostic Test (RDT) and Hemocue® test, respectively, were also considered in the analysis as potential aggravating factors of undernutrition. Malarial infection was confirmed by a positive RDT. Anemia was present when the Hemoglobin (Hb) level was under 10.0 grams per deciliter (g/dl) and was classified as severe when Hb < 7.0 g/dl, moderate when Hb was between 7.0 and 10.0 g/dl, and light when Hb between 10.0 and 11.0 g/dl.

### Analysis

A descriptive analysis of the data was performed to obtain households’, children’s, and mothers’ economic and sociodemographic characteristics. We then determined the levels of stunting, wasting, and underweight by computing the proportion of children classified according to the corresponding z-score on the total number of children as the denominator. We examined the coexistence of these three forms of undernutrition in the same children (both stunted and wasted, wasted and underweight, stunted and underweight) by crossing these abnormalities. Pearson’s chi-square (chi 2), Pearson’s r, and Cramer’s V correlation tests were performed to describe the bivariate relationship between the explanatory factors and childhood nutritional status. Statistical significance was set at P-value < 0.05. We used logistic regression models to assess the associations of stunting, wasting, and underweight with the explanatory factors. For that, the dependent variables were expressed as dichotomous according to the objective of assessing associated factors of undernutrition: category 0 (not stunted (>-2SD) and category 1 (stunted (>-2SD) for stunting, category 0 (not wasted (>-2SD) and category 1 (wasted (>-2SD) for wasting, and category 0 (not underweight (>-2SD) and category 1 (underweight (>-2SD) for underweight. After a full model with all the explanatory factors, each was analyzed using the LR-test. Significant factors with a *p-value* < 0.05 were considered at the end of these multivariable models. Odds ratios were provided with their 95% confidence intervals (95% CI). Afterwards, we applied the Pearson or Hosmer–Lemeshow goodness-of-fit test to examine if the models were correctly specified. The analysis was performed using Stata® version 17.0, StataCorp LLC, Texas, and Microsoft Excel 2010.

### Ethical standards disclosure

This study was conducted according to the guidelines laid down in the Declaration of Helsinki and all procedures involving research study participants were approved by the Ethical Committee of Heidelberg University (S-272/2013) and the National Ethics Committee of Burkina Faso (N° 2013-7-066). Written informed consent was obtained from all study participants before enrolment. Individual data from the study participants were anonymized before the analysis.

## Results

Data of 10,032 children aged 0–59 months were included in the analysis. 51% of children were male, and 93% lived in rural areas. 9.4% of children’s mothers were teenagers. Almost 91% of mothers did not have any formal education (Table 1).

**Table 1:** Sociodemographic and socioeconomic characteristics of children under five years of age and their mothers in Burkina Faso.

N = 10,032	Median (P25, P75)	n (%)		Median (P25, P75)	n (%)
**Sex**			**Distance health facility**	4.1 (1.8, 6.8)	
Male		5,080 (50.6)	< 2 km		2,656 (26.5)
Female		4,952 (49.4)	2–5 km		3,300 (32.9)
**Age (months)**	19 (9, 37)		> 5 km		4,076 (40.6)
[0–11]		3,042 (30.3)	**Climatic regions**		
[12–23]		2,872 (28.6)	Sahelian		3,468 (34.6)
[24–35]		1,234 (12.3)	Sudano-Sahelian		5,798 (57.8)
[36–47]		1,512 (15.1)	Sudanese		766 (7.6)
[48–59]		1,372 (13.7)	**Food security**		
**Residence**			Food security		2,884 (28.8)
Urban		740 (7.4)	Limit insecurity		2,943 (29.3)
Rural		9,292 (92.6)	Moderate insecurity		2,072 (20.7)
**Ethnic group**			Severe insecurity		2,133 (21.3)
Moose		6,491 (64.7)	**H Economic level** *		
Dioula & similar		1,676 (16.7)	1st quintile (poorest)		1,824 (18.2)
Fulani & similar		594 (5.9)	2^nd^ quintile (poor)		1,829 (18.2)
Others		1,271 (12.7)	3^rd^ quintile (middle)		1,975 (19.7)
**Number of children U5**	2 (1, 2)		4^th^ quintile (rich)		2,154 (21.5)
One		3,100 (30.9)	5^th^ quintile (richest)		2,250 (22.4)
Two		4,897 (48.8)	**Mother occupation**		
Three to six		2,035 (20.3)	Unoccupied		60 (0.8)
**Mother age (years)**	27 (23, 32)		Housewife		5,151 (69.4)
[15–19]		703 (9.4)	Regular work		2,208 (29.8)
[20–29]		3,902 (52.4)	**Mother educational level**		
[30–39]		2,365 (31.8)	None		6,793 (91.2)
[40–49]		477 (6.4)	Primary school		435 (5.8)
			Secondary & higher school		219 (2.9)

*According to standard multiple component analysis (MCA) method: housing (type of building, number of rooms, water, and energy supply), assets (TV, radio, fridge, etc.), houses and fields owned, and animals.

**Fig 1:**
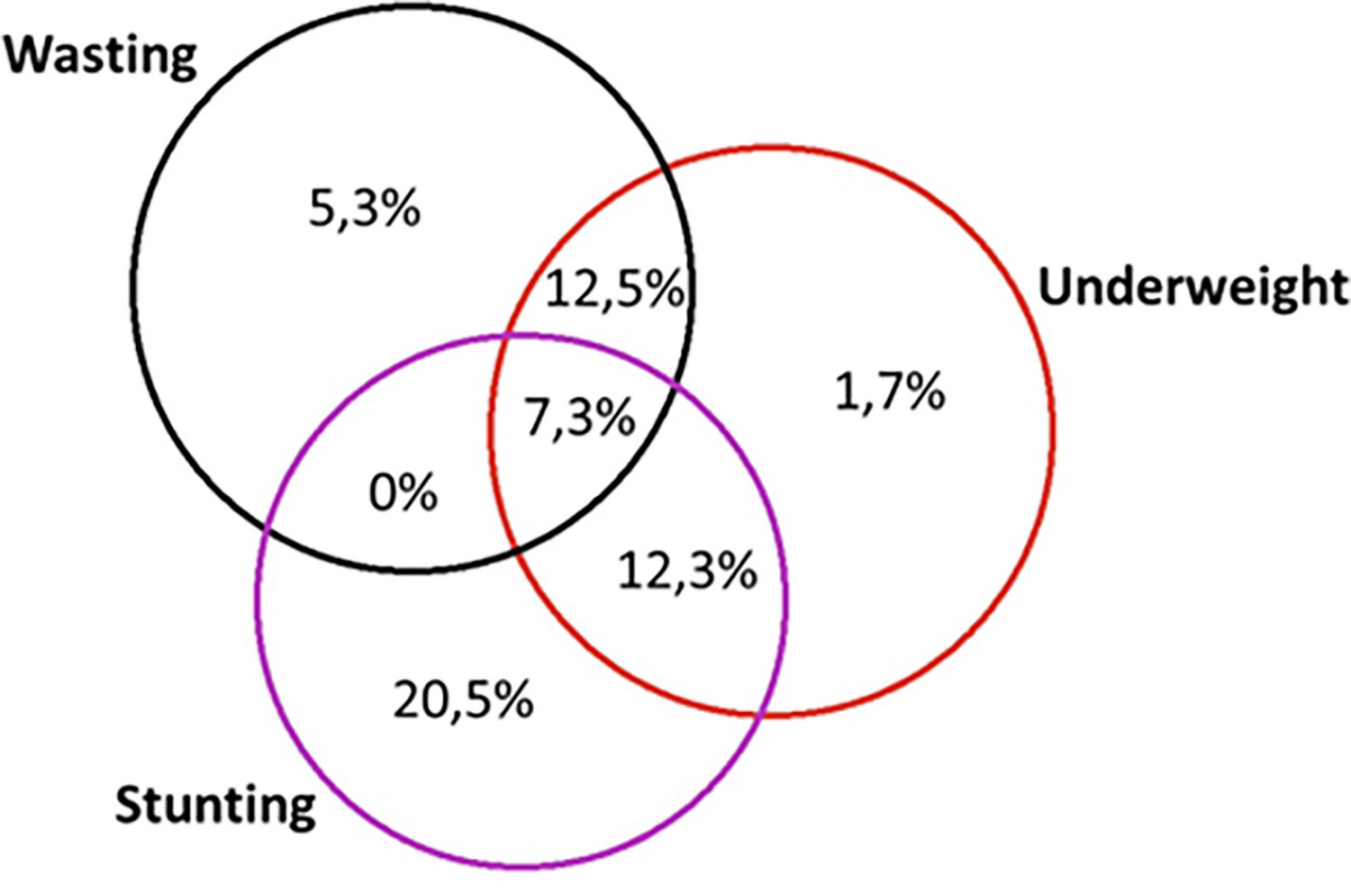
Co-existence of the 3 forms of undernutrition in children under 5 years in Burkina Faso.

Stunting was observed in 40,1% of children, with 21.5% being severe, wasting in 25.1%, with 14,5% severe, and underweight in 34%, with 16% severe. Anemia existed in 86.3% of children with 13% severe cases (Table 2). Among children with severe stunting, 14.8% had severe anemia, and 57.2% had moderate anemia. Among children with severe wasting, 13.6% and 54.3% had severe and moderate anemia, respectively. Among children with severe underweight, 15.3% had severe anemia, and 56.9% had moderate anemia. The crossing of the three forms of abnormalities saw eight categories: stunting & wasting & underweight 731 children (7.3%); both stunted and wasted 0, stunted and underweight 1,233 (12.3%); wasted and underweight 1,252 (12.5%); Stunting only 2059 (20.5%); Wasting only 535 (5.3%) Underweight only 174 (1.7%) and nourished 4,048 (40.5%) (Fig 1).

**Table 2:** Nutritional and health status of children under five years of age in Burkina Faso.

N = 10,032	Median (P25, P75)	n (%)	n (%)
**Height-for-age Z-score**	**- 1.6 (2.8, 0.5)**		
< - 3.0: Severe stunting		2,156 (21.5)	40.1
[-3.0; -2.0]: Moderate stunting		1,867 (18.6)	
≥ 2.0: Normal		6,009 (59.9)	59.9
**Weight-for-height Z-score**	**- 0.5 (- 2.0, 1.0)**		
< - 3.0: Severe wasting		1,453 (14.5)	25.1
[-3.0; -2.0]: Moderate wasting		1,065 (10.6)	
[-2.0; +2.0]: Normal		6,191 (61.7)	61.7
> +2.0: Overweight/ Obese		1,323 (13.2)	13.2
**Weight-for-age Z-score**	**- 1.2 (- 2.4, - 0.2)**		
< - 3.0: Severe underweight		1,632 (16.3)	33.8
[-3.0; -2.0]: Moderate underweight		1,758 (17.5)	
≥ 2.0: Normal		6,642 (66.2)	66.2
**Malaria RDT**			
Positive		3,818 (38.1)	
Negative		6,214 (61.9)	
**Hemoglobin (Hb) RDT**	**9.0 (7.7, 10.1)**		
< 7.0: Severe anemia		1,344 (13.4)	
7.0–9.9: Moderate anemia		5,598 (55.8)	
10.0–10.9: Light anemia		1,721 (17.2)	
≥ 11.0: No anemia		1,369 (13.7)	

The results of the logistic regression model showed that residence, maternal age, and mother’s education were not associated with stunting, wasting, or underweight. Table 3 reports the factors associated with stunting, wasting, and underweight in children under five years old in Burkina Faso. Overall, the sex of the child was significantly associated with stunting and being underweight, but not with wasting.

**Table 3:** Associated factors with underweight, stunting, wasting, and combination among children under five years in Burkina Faso, using multivariate logistic regression.

	Stunting			Wasting			Underweight			Stunting + Wasting + Underweight		
		Prevalence = 40.1%			Prevalence = 25.1%			Prevalence = 33.8%			Prevalence = 7.3%	
	%	AOR* (95% CI)	*p-value*	%	AOR (95% CI)	*p-value*	%	AOR (95% CI)	*p-value*	%	AOR (95% CI)	*p-value*
**Sex**			***< 0*.*001***			*0*.*14*			***< 0*.*001***			***< 0*.*001***
Female	42.9	1		25.9			36.0	1		8.4	1	
Male	37.4	0.79 (0.73–0.85)		24.4			31.7	0.82 (0.76–0.90)		6.2	0.73 (0.63–0.85)	
**Ethnic groups**			***< 0*.*001***			***< 0*.*001***			***0*.*03***			***0*.*01***
Moose	39.0	1		27.1	1		35.1	1		7.6	1	
Dioula & similar	40.1	0.78 (0.68–0.88)		19.5	1.20 (1.01–1.44)		28.9	1.02 (0.88–1.19)		5.4	1.07 (0.79–1.44)	
Fulani & similar	47.3	1.20 (1.01–1.42)		26.9	1.33 (1.10–1.63)		36.0	1.25 (1.04–1.50)		10.1	1.70 (1.26–2.28)	
Others	42.5	1.10 (0.96–1.24)		21.6	0.81 (0.69–0.95)		32.3	0.90 (0.78–1.04)		7.0	0.96 (0.74–1.23)	
**Economic level**			***0*.*005***			*0*.*12*			*0*.*06*			*0*.*06*
Richest	36.5	1		25.3			32.4			6.5		
Rich (4^th^ quintile)	40.8	1.18 (1.04–1.33)		25.3			35.3			7.4		
Middle	41.4	1.21 (1.07–1.37)		24.3			34.3			7.2		
Poor (2nd quintile)	40.8	1.18 (1.03–1.34)		25.7			33.6			7.4		
Poorest	41.7	1.25 (1.10–1.43)		24.8			33.4			8.0		
**Number of children U5 H** **			***0*.*004***			***< 0*.*001***			***< 0*.*001***			***0*.*003***
One	38.0	1		22.0	1		29.7	1		5.9	1	
Two	41.1	1.16 (1.05–1.27)		25.7	1.20 (1.08–1.34)		34.6	1.23 (1.12–1.36)		7.5	1.26 (0.88–1.31)	
Three and more	40.9	1.18 (1.05–1.33)		28.4	1.30 (1.32–1.48)		38.2	1.38 (1.22–1.55)		9.0	1.48 (1.19–1.83)	
**Distance health facility**			***0*.*005***			*0*.*76*			*0*.*20*			*0*.*16*
< 2 km	37.4	1		25.3			32.6			6.8		
2–5km	39.8	1.11 (1.01–1.23)		25.7			33.6			7.4		
> 5km	42.2	1.21 (1.10–1.35)		24.5			34.7			7.5		
**Climatic regions**			*0*.*28*			***< 0*.*001***			***< 0*.*001***			***< 0*.*001***
Sahelian	39.8			25.8	3.16 (2.44–4.08)		33.6	2.35 (1.89–2.92)		7.4	2.90 (1.86–4.52)	
Sudano-Sahelian	40.3			26.1	3.12 (2.34–4.16)		35.3	2.13 (1.67–2.72)		7.7	2.32 (1.41–3.81)	
Sudanese	40.3			14.6	1		23.2	1		3.8	1	
**Food security level**			***< 0*.*001***			***< 0*.*001***			***< 0*.*001***			***< 0*.*001***
Food security	40.9	1		30.4	1		38.1	1		8.8	1	
Limit insecurity	46.0	1.27 (1.13–1.42)		18.4	0.40 (0.33–0.50)		30.0	0.64 (0.53–0.77)		5.9	0.66 (0.46–0.93)	
Moderate insecurity	37.8	0.84 (0.74–0.95)		16.3	0.36 (0.31–0.41)		23.8	0.42 (0.36–0.47)		4.0	0.36 (0.27–0.46)	
Severe insecurity	33.1	0.67 (0.59–0.75)		35.8	1.10 (0.90–1.32)		42.9	1.12 (0.93–1.33)		10.4	1.26 (0.91–1.74)	
**Goodness of Fit Test**												
Hosmer–Lemeshow			*0*.*208*			*0*.*105*			*0*.*024*			*0*.*700*

* AOR: Adjusted Odds Ratio

** Number of children under five years of age in the household

Stunting was significantly associated with ethnic group, household economic level, number of children under five years of age in households, distance from household to health facility, and household food security. In addition to a higher prevalence (47.3%) compared to Moose (39.0%), stunting was associated with Fulani and similar ethnic groups with adjusted OR = 1.20 (95% CI: 1.01–1.42). The prevalence of stunting was higher in the rich (40.8%), poor (40.8%), middle (41.4%) and poorest (41.7%) households, compared to the richest category of household (36.5%). More, stunting was associated with these categories of economic level with Adj OR = 1.18 (95% CI: 1.04–1.33) for rich, Adj OR = 1.18 (95% CI: 1.03–1.34) for poor, Adj OR = 1.21 (95% CI: 1.07–1.73) for middle and Adj OR = 1.25 (95% CI: 1.10–1.43) for poorest. The prevalence of stunting was higher in households with at least two children (41.1%) compared to those with one child (38.0%) and stunting was associated with Adj OR = 1.16 (95% CI: 1.05–1.27). Compared to children who lived at a distance less than 2 km, those at more than 5 km suffered more from stunting which was associated with that with Adj OR = 1.21 (95% CI: 1.10–1.35). Households with limit food insecurity had a higher prevalence of stunting (46.0%) compared to those with food security (40.9%), while households with severe food insecurity had a lower prevalence of stunting (33.1%). More, stunting was associated with limit food insecurity with Adj OR = 1.27 (95% CI: 1.13–1.42) and with moderate and severe food insecurity with respectively Adj OR = 0.84 (95% CI: 0.74–0.95) and Adj OR = 0.67 (95% CI: 0.59–0.75).

Wasting was associated with ethnic group, number of children under five years in households, climatic conditions, and households’ food security. Compared to Moose (27.1%), the prevalence of wasting was low in Dioula and similar groups (19.5%) while wasting was associated with these ethnic groups with Adj OR = 1.20 (95% CI: 1.01–1.44) and with Fulani and similar groups with Adj OR = 1.33 (95% CI: 1.10–1.63). The prevalence of wasting was higher in households with three or more children (28.4%) compared to households with one child (22.0%) with association to that large number of children with Adj OR = 1.20 (95% CI: 1.08–1.34). The prevalence of wasting in children living in Sahelian and Sudano-Sahelian regions was higher (around 26%) than that in those living in the Sudanese region (14,6%). Furthermore, wasting was associated with these climatic conditions with Adj OR = 3.16 (95% CI: 2.44–4.08) and Adj OR = 3.12 (95% CI: 2.34–4.16) respectively. Children from severe food insecurity areas were more wasted 35.8% than those from security areas (30.4%). Unlike, those from limit insecurity areas 18.4% and moderate insecurity areas 16.3% were less wasted but wasting was associated with those with Adj OR = 0.40 (95% CI: 0.33–0.50) and Adj OR = 0.36 (95% CI: 0.31–0.41) respectively.

Being underweight was associated with ethnic group, number of children under five years in households, climatic conditions, and households’ food security. The prevalence of children suffering from underweight was lower in Dioula and similar groups 28.9% compared to Moose at 35.1%. Children in households with three or more children suffered from underweight (38.2%) more than with one child (29.7%). Underweight was associated with three or more children (Adj OR = 1.38 (95% CI: 1.22–1.55). The prevalence of underweight in children living in Sahelian (33.6%), and Sudano-Sahelian regions (35.3%) was higher than that in the Sudanese region (23,2%). Additionally, underweight was associated with these climatic conditions with Adj OR = 2.35 (95% CI: 1.89–2.92) and Adj OR = 2.13 (95% CI: 1.67–2.72) respectively. The prevalence of underweight was higher in children from severely insecure households 42.9% than those in security households (38.1%). Contrary, it was low in limit insecurity households (30.0%) and moderate insecurity (23.7%), and underweight was associated with those with respectively Adj OR = 0.64 (95% CI: 0.53–0.77) and Adj OR = 0.42 (95% CI: 0.36–0.47).

Suffering from both stunting and being underweight was associated with distance from the household to the health facility and household food security. The prevalence of children in both stunting and underweight was higher (13.5%) in households located at 5 km and more, compared to those at under 2 Km ((11.1%) and having both stunting and underweight was associated with living at 5 km and more, with Adj OR = 1.25 (95% CI: 1.07–1.46). Households with limited food insecurity (14.6%) had high prevalence of being both stunting and underweight adding an association with Adj OR = 1.18 (95% CI: 1.02–1.38). Also, being both stunting and underweight was associated with households in severe insecurity areas with Adj OR = 0.83 (95% CI: 0.69–0.99)).

For children carrying both wasting and underweight, the number of children under five of age in households, climatic regions, and the household food security were significantly associated. The prevalence of wasted and underweight children was greater in households with many children in households: two children 12.7% and three or more children 14.6% than in households with one child (10.7%). Suffering from both wasting and underweight was associated with those with Adj OR = 1.20 (95% 1.04–1.38) and (Adj OR = 1.30 (95%1.10–1.54) respectively. Sahelian and Sudano-Sahelian climatic regions presented high prevalence of both wasting and underweight (12.2% and 13.5% respectively) compared to Sudanese region (6.4%). Also, being both wasted and underweight was associated with these climatic conditions with Adj OR = 2.93 (95% CI: 2.14–4.01) and Adj OR = 3.20 (95% CI: 2.25–4.54) respectively. Households with limited insecurity (8.4%) and those with moderate insecurity (7.0%) were in low prevalence of having wasting and underweight in relation to those with food security (15.2%). However, being wasted and underweight was associated with living in households with limited insecurity with Adj OR = 0.40 (95% CI: 0.32–0.51) and in moderate insecurity with Adj OR = 0.35 (95% CI: 0.29–0.43). The children in severely insecure households had a higher prevalence of wasting added to being underweight (19.8%).

Stunting added to wasting and underweight in the same children was associated with ethnic group, the number of children under five in the household, climatic conditions, and food security. The prevalence was low in Dioula and similar groups (5.4%) but high in Fulani and similar groups (10.1%) compared to Moose. However, having both stunting, wasting and underweight was associated with being from Fulani and similar groups with Adj OR = 1.70 (95% CI: 1.26–2.28). Households with at least three children had a high prevalence of 9.0% than households with one child (5.9%) and being both stunted, wasted and underweight was associated with being from households with at least three children with Adj OR = 1.48 (95% CI: 1.19–1.83). Suffering from both stunting, wasting and underweight was associated with living in Sahelian (7.4%) with Adj OR = 2.90 (95% CI: 1.86–4.52) and in Sudano-Sahelian regions (7.7%) with Adj OR = 2.32 (95% CI: 1.41–3.81). Children from severe food insecurity households were more affected in 10.4% than those from security households (8.8%), unlike those from limit insecurity households 5.9% and moderate insecurity 4.0% were less affected. But being both stunted, wasted and underweight was associated with limit food insecurity with Adj OR = 0.66 (95% CI: 0.46–0.93) and with moderate food insecurity with Adj OR = 0.36 (95% CI:0.27–0.46).

## Discussion

Our study contributes to the existing evidence on the association of socio-demographic and economic factors with undernutrition in children under 5 in Burkina Faso, in the hope of informing action to combat malnutrition. Specifically, this study assessed the prevalence of certain indicators of undernutrition in under-5 years-old children in Burkina Faso and investigated household, parent, and child sociodemographic and economic factors associated with them.

We found a very high level of prevalence of wasting, stunting, and underweight with a very large difference in relation compared to the national averages for the same year and the trend in recent years (from 2009 to 2014: wasting 11.3% to 8.6%, underweight 26% to 20.1%, and stunting 35.1% to 29.1%) [36]. The difference was approximately 20% for wasting. Severe cases were equally high for each indicator in our study, with a similar difference to the national-level averages. The studied regions were chosen for inclusion in the PBF intervention because of their poor maternal and child indicators, which were reflected in our findings. In a broader regional comparison, our prevalence estimated were in the same range as those found in other sub-Saharan African settings [10–18].

Therefore, our results clearly demonstrated the need for action. To allow for targeted intervention planning, a good understanding of the factors contributing to the high prevalence of undernutrition is paramount. The second objective of our study was to contribute to the development of such an understanding. We identified the main associated factors: child sex (male), ethnic groups (Dioula and similar groups, and Fulani and similar groups), households’ low economic position, large families, long distance from households to health facilities, belonging to Sahelian and Sudano-Sahelian climatic regions, and belonging to households in a food insecurity zone. Other studied factors such as maternal age and school level did not emerge as important factors in our study, unlike in several prior studies in Sub-Saharan Africa [7, 15, 18, 37, 38], and neither did place of residence [12, 23, 37, 39–43].

Our study showed important differences in undernutrition prevalence among ethnic groups. This could be linked to household dietary practices, which differ by ethnicity, customs, prohibitions, and taboos, in instances depriving children, as well as pregnant and lactating women of good sources of protein and micronutrients. Children’s diet is subject to customary regulations in more ethnic contexts, with certain food prohibitions adding to parents’ poverty and childhood diseases [5].

A household’s low economic position increases the risk of children being stunted. Several studies in Sub-Saharan Africa have reported the same result [10, 12–14, 20, 37, 44–47]. In Burkina Faso, we have a context of poverty mainly in rural environments, with most households being farmers and breeders. Climatic and environmental conditions are often unfavorable to production and income-generating activities, leading to a disparity in the distribution of wealth in the country. Poverty affects a child’s nutritional status through insufficient food intake, exposure to infections, and a lack of basic health care such as vaccinations [7]. In addition, being from a large family with more than two children under five years of age increases the risk of being stunted or underweight, as in some studies in Sub-Saharan Africa and other low-income countries [19, 20, 48], as large households are unable to meet the nutritional needs of all family members. This underlines the need to sensitize households to multifaceted problems associated with their large size in the context of poverty.

Regarding access to health care, we found that distance was a predictive factor for stunting. In general, households are poorer and situated far from health facilities. In rural areas, most parents particularly mothers, do not have the means of transport at their disposal and need to travel on foot, or perhaps rarely by bicycle, resulting in general demotivation to visit health facilities to benefit from care, growth monitoring, or follow-up health education. Our findings underline the need to strengthen actions to significantly increase geographical healthcare coverage and reduce the average distance to less than 2 km.

The present study analyzed the prevalence and factors associated with the nutritional status of children under five years of age in Burkina Faso. However, our study had a few limitations. The overall sample is biased to include households with a higher proportion of women of reproductive age than would be observed in the population at large. Another limitation is the study’s reliance on secondary data, collected for a different primary aim, limiting the choice of theoretically relevant variables available for analysis, such as other healthcare indicators (diseases, diarrhea, etc.), other maternal factors such as marital status, mother’s parity, mother’s profession, mother’s nutritional status, and health characteristics. The employment situation of the mother existed in the database, but it only concerned employment during the thirty days prior to the survey. The others were incompletely filled in the database when they were collected. Explanatory factors such as the distribution of children according to households’ affiliation to the country’s climatic areas and according to the national distribution of households for food insecurity, were additionally created from the national reported results but not collected during the study. Another limitation of this study would be the non-application of analysis in relation to the hierarchical structure of the data by employing multilevel binary logistic regression models. The study used extracted data of children following criteria of completeness and plausibility for all relevant variables for analysis according to the objectives. That did not take this hierarchical structure into account during extraction. Then, it would be inappropriate to apply multilevel binary logistic regression models.

## Conclusion

The current study aimed to provide information about children under five years of age’ nutritional status (undernutrition) and the determinants at the individual and community levels. Despite a few limitations, this study highlighted the children and maternal, household, and environmental factors associated with childhood undernutrition (stunting, wasting, and underweight) in Burkina Faso. The results showed that the prevalence of stunting, wasting, and underweight was high and constituted an important health concern in children under five years in this country. Over and above the child’s demographic characteristics, particularly their sex, key determinants of undernutrition in Burkina Faso found in this study were ethnic groups, household wealth status, number of children under five years, and the distance from household to health facility. The positive or negative influence of these factors should be considered when developing strategies and interventions to reduce nutritional problems in children under five years. In this regard, climatic conditions and household food security should be considered. The results of our study indicate the need to improve both health and economic levels of the community and households by developing social and health programs for parents to raise awareness of existing nutritional problems with their factors and combat them, and by strengthening new coping and resilience systems for households’ productivity.

## Supporting information

S1 Data Study database from the baseline survey of the impact evaluation of the PBF program in Burkina Faso. (DTA)
